# NOA61-Polymer Fiber Fizeau Interferometer with a Flexible NOA65-Polymer Taper for Simultaneous Measurement of Tilt Angle and Temperature

**DOI:** 10.3390/polym13162798

**Published:** 2021-08-20

**Authors:** Cheng-Ling Lee, Chi-Shiang Chen, Chun-Ren Yang, Rui-Cheng Zeng

**Affiliations:** Department of Electro-Optical Engineering, National United University, No. 2 Lien-Da, Miaoli City 36003, Taiwan; U0723013@gm.nuu.edu.tw (C.-S.C.); alanboy927@gmail.com (C.-R.Y.); watermelon5424@gmail.com (R.-C.Z.)

**Keywords:** fiber tilt sensor, NOA61, NOA65, polymer, simultaneous measurement, fiber Fizeau interferometer, taper

## Abstract

This study proposes and experimentally demonstrates a NOA61-polymer fiber Fizeau interferometer (PFFI) connected to a flexible NOA65-polymer taper (PT) for simultaneous measurement of tilt angle and temperature (T). The PT/PFFI fiber sensor consists of a taper-shaped flexible NOA65 polymer and single-mode fiber with an endface that is attached to a NOA61-polymer. The NOA61-polymer of PFFI is highly sensitive to variations of T with high repeatability and enables the simultaneous measurement of tilt angle by connecting with the highly flexible NOA65-PT. the interference fringe visibility of optical spectra in the PFFI can be highly controlled by the tilt angle of the PT and is thus capable of measuring tilt angles with high sensitivity. On the other hand, wavelength shifts of the spectra in the PFFI only occur when T varies. The proposed PT/PFFI can simultaneously detect the tilt state and the variation of surrounding T by measuring the optical spectral responses and eliminating cross sensitivity. Experimental results demonstrate the PT/PFFI can simultaneously measure tilt angles and T with good sensitivities and obtain averages of 0.4 dB/° and 0.17 nm/°C, respectively.

## 1. Introduction

Distributed and multiplexed capability of fiber optic sensors are the most important advantages that can integrate various passive optics sensors for multiple parameters and region-sensing applications. Therefore, advanced fiber optic sensors with the ability of simultaneously sensing multi-parameters have recently received much attention. These sensors can simultaneously measure any of the following parameters, pressure [1], temperature [1,2,3,4,5,6,7,8,9,10,11,12,13,14,15,16], displacement [2], strain [3,4,8], tilt angle [13,14,15,16], refractive index (RI) [5,6,7,9,11] and humidity [10,12]. Among the sensing parameters mentioned above, the tilt angle (ϕ) of structure and temperature (T) of the surroundings have important utility values in green buildings and health bridges, and therefore, attract much research interest. In achieving multiplex sensing capability, the key issue for practical applications of fiber optic sensors is not only cross sensitivity but also the complexity of sensing configurations. Thus far, several fiber optical sensors for simultaneous measurement of the tilt angle and temperature are proposed and investigated, but most are incorporated with well-known fiber Bragg gratings (FBG)-based devices [13,14,15]. For example, a simple fiber optic inclinometer is developed based on linearly chirped FBG written in both fused taper transitions [13]. Similarly, the tilt fiber sensor based on a taper-shaped polymer incorporating a FBG is proposed [14]. Simultaneous measurement of 2D tilt angles and temperature by a fiber optic sensor is also proposed and experimentally demonstrated. The sensing head consists of two FBGs to achieve various sensing performances [15]. The experimental results of the above tilt angle fiber sensors show that their proposed sensing configuration responds well to the tilt angles, but the sensitivity and resolution of T responses may not be sufficiently recognizable due to the low thermal expansion coefficient of the fused fibers. In 2016, Feng et al. reported a fiber inclinometer that consists of a micro-fiber taper followed by an air-gap microcavity. The fringe contrast of the interferometer is highly sensitive to fiber bending and is thus capable of measuring tilt angles. However, the proposed fiber sensor cannot simultaneously measure T, to which the fused silica fiber is T insensitive [16]. In 2020, a novel, highly sensitive, and simple structure based on a tapered polymer as a tilt fiber sensor is developed to sensitively measure tilt angles [17]. The fiber-optic tilt sensor consists of a tapered polymer fabricated by a flexible adhesive NOA65-polymer with low modulus (20,000 psi) from Norland Products Inc. (Cranbury, NJ, USA) [18]. The NOA65-polymer taper easily generates a bend in the fiber sensor that is strongly correlated with tilt angles. Experimental results of the above sensors show that the sensing configuration responds well to the tilt angles, but the sensitivity and resolution of the T responses may be not sufficiently recognizable.

In this study, we connect a T-sensitive NOA61-polymer fiber Fizeau interferometer (PFFI) with a flexible adhesive NOA65-polymer taper (PT) for simultaneous sensing of tilt (ϕ) and temperature (T). The proposed PT/PFFI consists of a tapered polymer made with NOA65 and the endface of a single mode fiber (SMF) attached to a UV-cured NOA61-polymer to form an ultracompact microcavity. The NOA61-polymer in the PFFI is highly sensitive to T variations with high repeatability, and achieve an instantaneous measurement of ϕ by connecting a flexible NOA65-PT [19]. The NOA65-PT polymer is also T-sensitive with a thermal expansion coefficient (TEC) of 2.2 × 10^−4^ (°C^−1^) is similar to that of the NOA61 [20]. However, the NOA65-PT is the part of the inclinometer that merely controls the light into the NOA61-PFFI for creating interference. Therefore, the thermal expansion effect on the NOA65 is ignorable for the optical interference from NOA61-PFFI. Variation of fringe visibility (FV) and wavelength shifts (Δλ) of the spectral interference of the sensor correspond to responses of the ϕ and T, respectively. The interference fringe always remains unshifted during the fiber tilts, but optical power and FV showed considerable changes. On the other hand, the interference fringe shifts when T varies while the optical power of reflection is almost unchanged at a fixed ϕ. Thus, the proposed PT/PFFI fiber sensor can detect the tilt states and discriminate the variation of surrounding T by monitoring the FV and Δλ removing the cross-sensitivity of ϕ and T. The experimental results demonstrate that the developed sensor can measure ϕ and T simultaneously with good measurement sensitivities and averages of 0.4 dB/° and 0.17 nm/°C, respectively.

## 2. Sensor Fabrication and Principle

**Figure 1:**
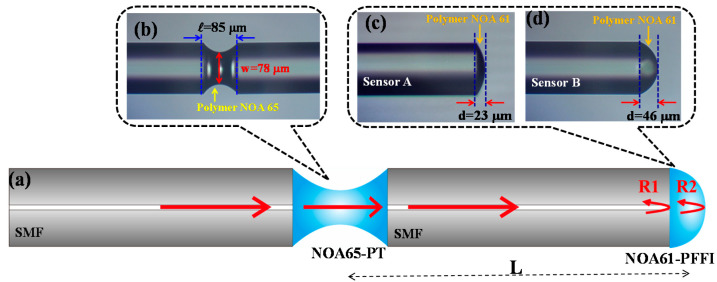
(**a**) Configuration of the proposed sensor with (**b**) taper-shaped NOA65-polymer with L/d = 85 μm/78 μm and (**c**) sensor A with d = 23 μm or (**d**) sensor B with d = 46 μm NOA61-PFFI.

The configuration of the PT/PFFI sensor is based on a PFFI formed by a T-sensitivity NOA61-polymer combined with a NOA65-polymer taper (PT) with good flexibility, as presented in Figure 1.

**Figure 2:**
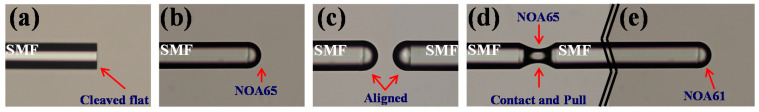
Fabrication of the PT/PFFI by the processes of (**a**) cleaving fiber flat, (**b**) NOA65 attached to fiber endface, (**c**) two fiber-NOA65 aligned, (**d**) contact and pull into a tapered shape to form the NOA65-PT, and (**e**) sensor endface with NOA61 attached to form the NOA61-PFFI.

The taper-shaped NOA65 polymer is the main part of the inclinometer that controls the light into the NOA61-PFFI for generating low-finesse interference. Here, the NOA65 (n_D_ = 1.52) [18,19] polymer materials used are based on a type of optical adhesive, ultraviolet (UV)-cured polymer, with good elongation and is more elastic than conventional fiber taper-based tilt sensors [14,15]. In addition, the NOA61-polymer with the high refractive index of n_D_ = 1.56 can produce high FV that is more suitable for this study [10]. The fabrication is monitored by a charge-coupled device (CCD) microscope. Figure 2 shows the fabrication steps of the PFFI combined with PT. The NOA65-polymer taper is simply fabricated with the assistance of three-axis translation stages at exact alignment [17]. After accomplishing the desired taper shape of the PT, the monitored translation stages are used to attach a thick film of NOA61-polymer onto the endface of the SMF (Corning^@^ SMF-28e) to form the PFFI (Figure 2e). The thickness of NOA61 can be carefully controlled by the number of attachment times, as plotted in Figure 2c,d. Here, Figure 1b shows the structure of the NOA65-PT with the tapered region (L)/waist diameter (w) of L/w = 85 μm/78 μm and Figure 1c,d shows the NOA61-PFFI with a cavity length of d = 23 and 46 μm for sensor A and B, respectively.

**Figure 3:**
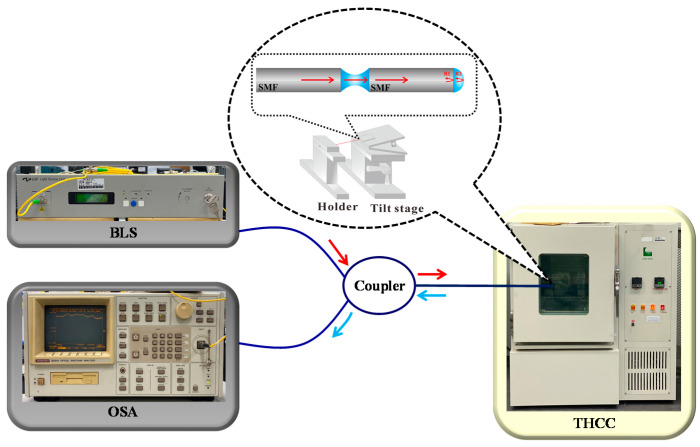
Experimental setup for simultaneously measuring ϕ and T.

Figure 1a also illustrates the principle of the proposed PT/PFFI. When the NOA65-PT is bent due to the tilt, light propagating into the NOA61-PFFI intensely decreases with deviation from the center of the fiber axis to instantly weaken the FV of the optical interferences. Figure 3 displays the experimental setup with a broadband light source (BLS, BLS-GIP Technology) and a 2 × 1 optical coupler, which reflects off the endfaces of the PFFI, and returns to the coupler. Finally, the spectral response readouts are directly measured by an optical spectrum analyzer (OSA, Advantest Q8381 A). The PT/PFFI is located inside a temperature and humidity controlling chamber (THCC, LABSON, No. LA-85R) for varying the T and ϕ with fixed humidity.

**Figure 4:**
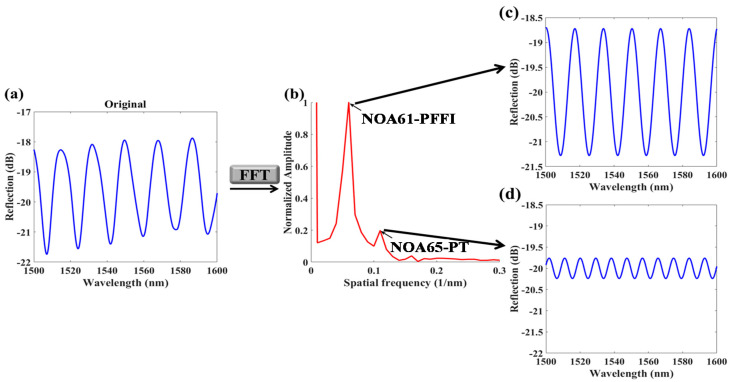
(**a**) Optical response of superimposed interference measured by the OSA; (**b**) superimposed spectra processed by FFT; separated optical spectra of (**c**) NOA61-PFFI and (**d**) NOA65-PT.

The used THCC with temperature (T) accuracy of ~0.2 °C and relative humidity (RH) accuracy of ~2% that are applied to control the T and RH. Since the used polymer device can affected by the surrounding humidity [10], all the measurements are accomplished under a fixed RH = 50%. Therefore, reactions of tilt sensing results can be readily obtained by monitoring FV values or optical power of the reflective interference only from signals of the NOA61-PFFI. If the proposed sensor tilts at a fixed T, then the interference power and FV can considerably decay. Moreover, when the sensor is under T variation at a fixed ϕ, the interference spectra are red-shifted as T increases and are blue-shifted as T decreases. In the reflection spectra, interference signals of two cavities of the NOA65-PT and NOA61-PFFI are superimposed and collected by the OSA. However, the interference signal of NOA65-PT is much weaker than that of the NOA61-PFFI. Analyses of the optical responses from the combined interferences are accomplished using the fast Fourier transform (FFT) method, which is used to separate multiple interferences in spatial frequency into two individual spatial frequencies for the NOA65-PT and NOA61-PFFI. Figure 4 shows the optical responses of the superimposed interference, separating into the spectra through the simple FFT method. The superimposed interference plotted in Figure 4a is processed by the FFT to obtain the spatial frequency spectra, as shown in Figure 4b, indicating that the signal of NOA61-PFFI is higher than that of the NOA65-PT. Subsequently, the spatial frequencies for the NOA65-PT and NOA61-PFFI can be individually separated by the inverse FFT with signal processing, as plotted in Figure 4c,d respectively. In this study, only the interference signals of NOA61-PFFI must be measured to achieve multiple parameters sensing.

## 3. Experimental Results and Discussion

**Figure 5:**
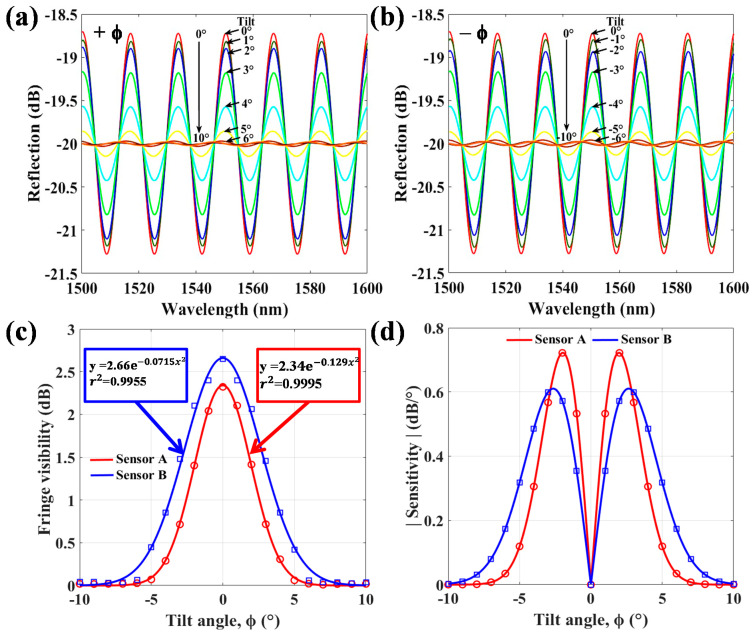
Optical responses for the variations of tilt angle (ϕ) by (**a**) positive ϕ and (**b**) negative ϕ; (**c**) Responses of FV to tilt angles by sensor A with d = 23 μm and sensor B with d = 46 μm; (**d**) ϕ-related sensitivities of the tapered polymer sensors for sensors A and B.

In the experiment, the proposed PT/PFFI device is placed inside the THCC, a closed space in which the tilt angle is operated from ϕ = –10°~+10° with a step of 1° under fixed T and fixed relative humidity of 50%. Figure 5 shows the results. For the sensor with d = 46 μm, Figure 5a,b show the spectral responses to positive ϕ and negative ϕ, respectively. These experimental results are almost similar due to the central symmetry of the PT structure. Figure 5c displays variations of FV of reflection for sensors A and B as the ϕ changes from −10°~+10°. When the ϕ of the sensor increases, the FV has high attenuation. Moreover, at high angles, the FV gradually weakens and then vanishes because the optical light almost leaks out in the great bending of PT. When different sensing structures of sensors A and B are used (as shown in Figure 1), the responses and sensitivities of ϕ variation from −10° ~ +10° are obtained and shown in Figure 5c,d, respectively. Sensor A (d = 23 μm) with thinner PT seems to be more sensitive than sensor B (d = 46 μm). However, the tilt measurement range is relatively small for sensor A. The tilt sensitivity S is defined as:

(1) $$S=\frac{d\left(\mathrm{FV}\right)}{d\phi }$$

**Figure 6:**
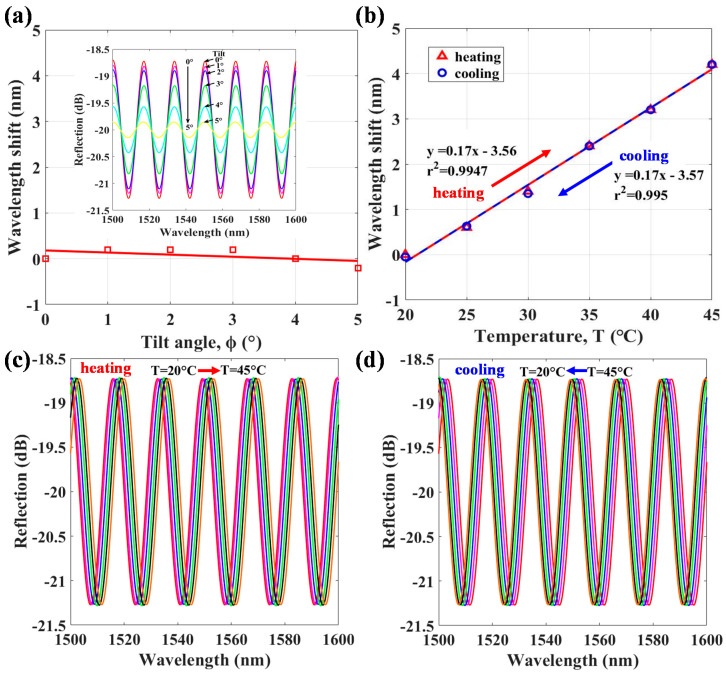
Wavelength shift responses for the variations of (**a**) tilt angle, ϕ at fixed T = 25 °C and (**b**) temperature, T by (**c**) heating and (**d**) cooling with fixed ϕ = 0°.

Figure 5d shows that high responses are achieved from tilt angles of ϕ = −10° ~ +10° and sensors A and B obtain the highest sensitivities of 0.72 dB/° at ϕ of ±2° and 0.6 dB/° at ϕ of ±2.7°, respectively. It is worth mentioning that in the tilt sensing, the interference power and FV highly vary but the wavelength peaks of optical interferences are nearly unshifted, as shown in Figure 6a. The inset of Figure 6a displays the detailed optical interference spectra for the variations of corresponding parameters ϕ at T = 25 °C. On the other hand, when the sensor is heating and cooling with T, the range is 20 ~ 45 °C at a fixed ϕ = 0°. The interference is red-shifted as T increases and vice versa. Figure 6b plots the T-sensitivity of the proposed PT/PFFI sensor. The detailed optical interference spectra for heating and cooling are shown in the Figure 6c,d, respectively. The results indicate that the optical responses have high repeatability, and the sensitivity is linearly proportional to T with 0.17 nm/°C. The obtained results of the T sensitivity may not be high; however, the optical power of the spectral responses almost does not decay that can easily distinguish non-tilt variations. Based on the above sensing results, the proposed PT/PFFI can demonstrate the superiority of sensing characteristics to avoid the cross-sensitivity of the measured parameters.

**Figure 7:**
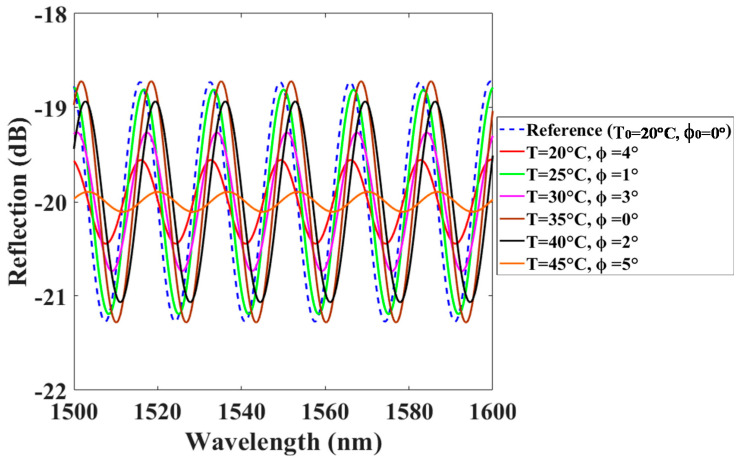
Interference spectra of the sensor when ϕ and T simultaneously change at different conditions.

**Figure 8:**
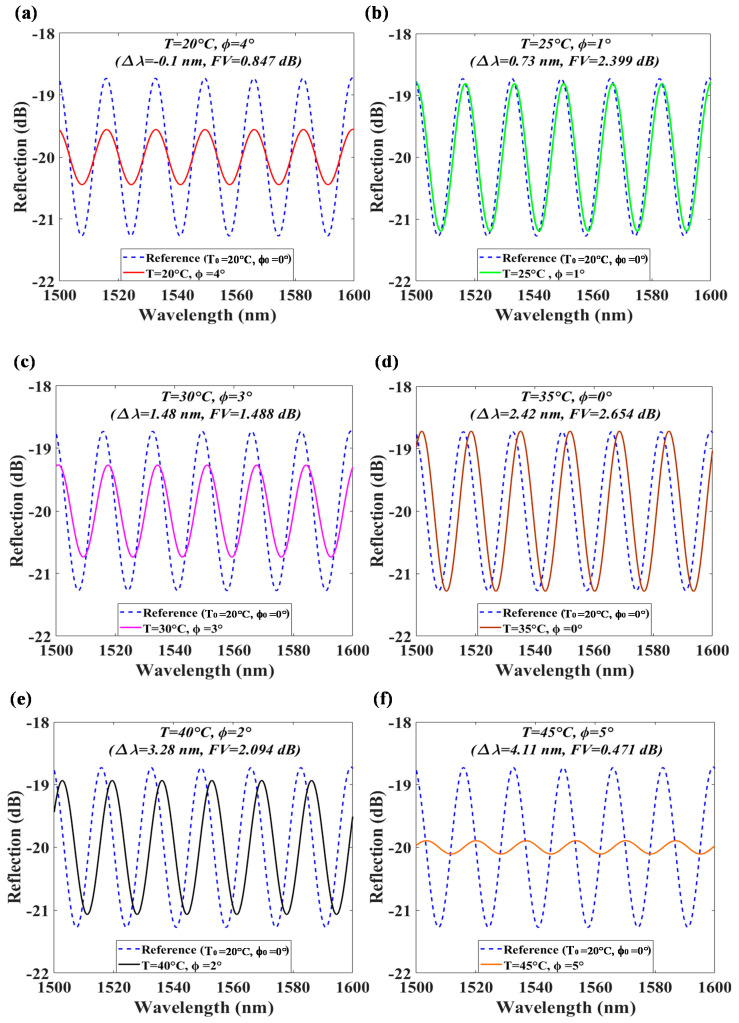
Interference spectra individually compared with the reference spectrum for (**a**) T = 20 °C, ϕ = 4°; (**b**) T = 25 °C, ϕ = 1°; (**c**) T = 30 °C, ϕ = 2°; (**d**) T = 35 °C, ϕ = 0°; (**e**) T = 40 °C, ϕ = 2°; and (**f**) T = 45 °C, ϕ = 5°.

The effectiveness for the developed sensing configuration is examined by simultaneously varying ϕ and T from their reference values of ambient (set as ϕ_0_ = 0° and T_0_ = 20 °C) to various conditions, as shown in Figure 7 and Figure 8, respectively. The optical spectra of the initial condition of ϕ_0_ = 0° and T_0_ = 20 °C as the reference for the test sensor B are recorded at the beginning of the experimental investigations (as blue-dashed lines of Figure 7 and Figure 8). Figure 7 presents the simultaneous measurement of the arbitrarily chosen ϕ and T for many cases. Clearly, the variation of the FV is only due to changes of ϕ and the wavelength shift (Δλ) is merely affected by T variation. The parameters of ϕ and T can be respectively estimated by using the experimental relations of FV = 2.66⋅exp(−0.0715⋅ϕ^2^) and ∆λ = 0.17⋅T − 3.565 when these two factors are measured.

Figure 8 shows the individual interference spectra of every case to compare with that of the initial ϕ_0_ and T_0_. The arbitrary conditions are: (a) T = 20 °C, ϕ = 4°; (b) T = 25 °C, ϕ = 1°; (c) T = 30 °C, ϕ = 2°; (d) T = 35 °C, ϕ = 0°; (e) T = 40 °C, ϕ = 2°; and (f) T = 45 °C, ϕ = 5°. The measured values of the ϕ and T can be estimated by the measured FV and Δλ in the optical interference spectra by using the experimental relations of FV = 2.66⋅exp(−0.0715⋅ϕ^2^) and ∆λ = 0.17⋅T − 3.56. The measured FV and ∆λ of each case are: (a) Δλ = −0.1 nm, FV = 0.847 dB (b) Δλ = 0.73 nm, FV = 2.399 dB, (c) Δλ = 1.48 nm, FV = 1.488 dB, (d) Δλ = 2.42 nm, FV = 2.654 dB, (e) Δλ = 3.28 nm, FV = 2.094 dB, and (f) Δλ = 4.11 nm, FV = 0.471 dB for determining every case of the measured T and ϕ, as shown in Figure 8 and also listed in the following Table 1. Here, the second decimal point for the measured Δλ is an artificial estimation value, so that the accuracy of the measured Δλ as well as the obtained T is one decimal point.

**Figure 9:**
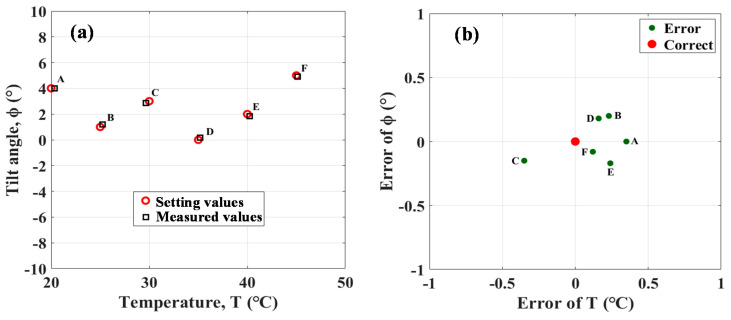
(**a**) Sample data for measurement comparison; (**b**) Error analysis of the sample measurement.

The results in Figure 7 and Figure 8 and Table 1 show that the simultaneous sensing of ϕ and T is accomplished. The simultaneously measured values of ϕ and T are very close to the setting values displayed in the THCC and tilt stages. The six setting conditions of (a) T = 20 °C, ϕ = 4°; (b) T = 25 °C, ϕ = 1°; (c) T = 30 °C, ϕ = 2°; (d) T = 35 °C, ϕ = 0°; (e) T = 40 °C, ϕ = 2°; and (f) T = 45 °C, ϕ = 5° can achieve the measured T and ϕ with (a) T = 20.38 °C, ϕ = 4°; (b) T = 25.26 °C, ϕ = 1.2°; (c) T = 29.68 °C, ϕ = 2.85°; (d) T = 35.21 °C, ϕ = 0.18°; (e) T = 40.26 °C, ϕ = 1.83°; and (f) T = 45.09 °C, ϕ = 4.92°, respectively. The measured errors in T and ϕ are also shown in Figure 9 with (a) T_error_ = 0.38 °C, ϕ_error_ = 0°; (b) T_error_ = 0.26 °C, ϕ_error_ = 0.2°; (c) T_error_ = −0.32 °C, ϕ_error_ = −0.15°; (d) T_error_ = 0.21 °C, ϕ_error_ = 0.18°; (e) T_error_ = 0.26 °C, ϕ_error_ = −0.17°; and (f) T_error_ = 0.09 °C, ϕ_error_ = −0.08°, respectively. Based on the above data, the average errors of the measured ϕ and T of the six simultaneous measurements are approximately 0.253 °C and 0.13°, respectively. The above results demonstrate the effectiveness of the proposed PT/PFFI sensor for simultaneous measurement of ϕ and T. The small errors of the measurements are attributable to the deviations of operating the instruments. Moreover, non-flat endface of the fibers caus20ed by the mechanical fiber cleaver is another reason. The flat surface would be effectively cleaved by using a femtosecond laser to improve the measured accuracy [21].

## 4. Conclusions

This study proposes a new sensing configuration combining the high T sensitivity and repeatability NOA61-PFFI to the flexible NOA65-PT. Thus, the sensor can achieve the simultaneous measurement of ϕ and T. The experimental results show that the interference FV of the optical spectra is only correlated with ϕ and the wavelength shifts (Δλ) of the interference spectra are only affected by T variations. Consistent results illustrate that by using the proposed sensing scheme with the specific characteristics of the used materials (NOA61 and NOA65 polymers), the fiber device can simultaneously and effectively measure T and ϕ with small errors. Furthermore, the proposed PT/PFFI can independently discriminate the tilt angle state and the surrounding T variations from the measured spectra. The sensing results demonstrate that the PT/PFFI can measure ϕ and T simultaneously with good sensitivities and averages of 0.4 dB/° and 0.17 nm/°C, respectively. The obtained results of the T and ϕ sensitivities may not be high but are comparable with those based on the FBG sensors, however, the advantages of this solution are that it is simple, flexible, easy to fabricate, and does not require an expansive laser system for writing the FBGs.

## Figures and Tables

**Table 1:** Evaluating the simultaneous measurement of ϕ and T in different conditions by measuring the ∆λ and FV.

Measurement	Setting Conditions					
	(a) T = 20 °C, ϕ = 4°	(b) T = 25 °C, ϕ = 1°	(c) T = 30 °C, ϕ = 3°	(d) T = 35 °C, ϕ = 0°	(e) T = 40 °C, ϕ = 2°	(f) T = 45 °C, ϕ = 5°
Measured Δλ (nm)	−0.1	0.73	1.48	2.42	3.28	4.1
Measured FV (dB)	0.847	2.399	1.488	2.654	2.094	0.471
Measured T (°C), ϕ (°)	T = 20.38, ϕ = 4	T = 25.26, ϕ = 1.2	T = 29.68, ϕ = 2.85	T = 35.21, ϕ = 0.18	T = 40.26, ϕ = 1.83	T = 45.09, ϕ = 4.92
Measured errors of T (°C), ϕ (°)	T_error_ = 0.38, ϕ_error_ = 0	T_error_ = 0.26, ϕ_error_ = 0.2	T_error_ = −0.32, ϕ_error_ = −0.15	T_error_ = 0.21, ϕ_error_ = 0.18	T_error_ = 0.26, ϕ_error_ = −0.17	T_error_ = 0.09, ϕ_error_ = −0.08
